# A framework of biomarkers for brain aging: a consensus statement by the Aging Biomarker Consortium

**DOI:** 10.1093/lifemedi/lnad017

**Published:** 2023-05-06

**Authors:** Aging Biomarker Consortium, Yu-Juan Jia, Jun Wang, Jun-Rong Ren, Piu Chan, Shengdi Chen, Xiao-Chun Chen, Jagadish K Chhetri, Junhong Guo, Qihao Guo, Lingjing Jin, Qiang Liu, Qiang Liu, Wenlin Ma, Zhiyong Mao, Moshi Song, Weihong Song, Yi Tang, Difei Wang, Peijun Wang, Lize Xiong, Keqiang Ye, Junjian Zhang, Weiqi Zhang, Xiaoqing Zhang, Yunwu Zhang, Zhanjun Zhang, Zhuohua Zhang, Jialin Zheng, Guang-Hui Liu, Yi Eve Sun, Yan-Jiang Wang, Gang Pei

**Affiliations:** Department of Neurology and Centre for Clinical Neuroscience, Daping Hospital, Third Military Medical University, Chongqing 400042, China; Department of Neurology, First Affiliated Hospital, Shanxi Medical University, Taiyuan 030001, China; Department of Neurology and Centre for Clinical Neuroscience, Daping Hospital, Third Military Medical University, Chongqing 400042, China; Department of Neurology and Centre for Clinical Neuroscience, Daping Hospital, Third Military Medical University, Chongqing 400042, China; National Clinical Research Center for Geriatric Diseases, Xuanwu Hospital, Capital Medical University, Beijing 100053, China; Department of Neurology and Institute of Neurology, Ruijin Hospital, Shanghai Jiao Tong University School of Medicine, Shanghai 200025, China; Department of Neurology, Union Hospital of Fujian Medical University, Fuzhou 350001, China; National Clinical Research Center for Geriatric Diseases, Xuanwu Hospital, Capital Medical University, Beijing 100053, China; Department of Neurology, First Affiliated Hospital, Shanxi Medical University, Taiyuan 030001, China; Department of Gerontology, Shanghai Sixth People’s Hospital Affiliated to Shanghai Jiao Tong University School of Medicine, Shanghai 200233, China; Department of Neurology and Neurological Rehabilitation, Shanghai Disabled Persons’ Federation Key Laboratory of Intelligent Rehabilitation Assistive Devices and Technologies, Yangzhi Rehabilitation Hospital (Shanghai Sunshine Rehabilitation Center), Tongji University, School of Medicine, Shanghai 200092, China; Institute on Aging and Brain Disorders, The First Affiliated Hospital of USTC, Hefei National Laboratory for Physical Sciences at the Microscale, Division of Life Sciences and Medicine, University of Science and Technology of China, Hefei 230026, China; Department of Neurology, Institute of Neuroimmunology, Tianjin Medical University General Hospital, Tianjin 300052, China; Department of Cardiology, Shanghai Tongji Hospital, Tongji University School of Medicine, Shanghai 200092, China; Shanghai Key Laboratory of Maternal Fetal Medicine, Clinical and Translational Research Center of Shanghai First Maternity and Infant Hospital, Frontier Science Center for Stem Cell Research, Shanghai Key Laboratory of Signaling and Disease Research, School of Life Sciences and Technology, Tongji University, Shanghai 200092, China; State Key Laboratory of Membrane Biology, Institute of Zoology, Chinese Academy of Sciences, Beijing 100101, China; Institute of Aging, Key Laboratory of Alzheimer’s Disease of Zhejiang Province, Zhejiang Clinical Research Center for Mental Disorders, School of Mental Health and The Affiliated Kangning Hospital, Wenzhou Medical University, Oujiang Laboratory, Wenzhou, Zhejiang 325035, China; Department of Neurology & Innovation Center for Neurological Disorders, Xuanwu Hospital, Capital Medical University, National Center for Neurological Disorders, Beijing 100053, China; Department of Gerontology, Shengjing Hospital of China Medical University, Shenyang 110000, China; Department of Radiology, Tongji Hospital, Shanghai Frontiers Science Center of Nanocatalytic Medicine, The Institute for Biomedical Engineering & Nano Science, School of Medicine, Tongji University, Shanghai 200092, China; Shanghai Key Laboratory of Anesthesiology and Brain Functional Modulation, Translational Research Institute of Brain and Brain-Like Intelligence, Shanghai Fourth People’s Hospital, Tongji University, Shanghai 200434, China; Faculty of Life and Health Sciences, Shenzhen Institute of Advanced Technology, Chinese Academy of Science, Shenzhen 518055, China; Department of Neurology, Zhongnan Hospital of Wuhan University, Wuhan 430071, China; CAS Key Laboratory of Genomic and Precision Medicine, Beijing Institute of Genomics, Chinese Academy of Sciences and China National Center for Bioinformation, Beijing 100101, China; Translational Medical Center for Stem Cell Therapy, Shanghai East Hospital, School of Medicine, Tongji University, Shanghai 200120, China; Fujian Provincial Key Laboratory of Neurodegenerative Disease and Aging Research, Institute of Neuroscience, School of Medicine, Xiamen University, Xiamen 361102, China; State Key Laboratory of Cognitive Neuroscience and Learning, Beijing Normal University, Beijing 100875, China; Hunan Key Laboratory of Molecular Precision Medicine, Department of Critical Care Medicine, Xiangya Hospital, Central South University, Changsha 410008, China; Center for Translational Neurodegeneration and Regenerative Therapy, Tongji Hospital affiliated to Tongji University School of Medicine, Shanghai 200072, China; University of Chinese Academy of Sciences, Beijing 100049, China; State Key Laboratory of Membrane Biology, Institute of Zoology, Chinese Academy of Sciences, Beijing 100101, China; Institute for Stem Cell and Regeneration, Chinese Academy of Sciences, Beijing 100101, China; Stem Cell Translational Research Center, Tongji Hospital, Tongji University School of Medicine, Shanghai 200065, China; Department of Neurology and Center for Clinical Neuroscience, Daping Hospital, Third Military Medical University, Chongqing 400042, China; Key Laboratory of Ageing and Brain Disease, Chongqing 400042, China; Collaborative Innovation Center for Brain Science, School of Life Science and Technology, Tongji University, Shanghai 200092, China

## Abstract

China and the world are facing severe population aging and an increasing burden of age-related diseases. Aging of the brain causes major age-related brain diseases, such as neurodegenerative diseases and stroke. Identifying biomarkers for the effective assessment of brain aging and establishing a brain aging assessment system could facilitate the development of brain aging intervention strategies and the effective prevention and treatment of aging-related brain diseases. Thus, experts from the Aging Biomarker Consortium (ABC) have combined the latest research results and practical experience to recommend brain aging biomarkers and form an expert consensus, aiming to provide a basis for assessing the degree of brain aging and conducting brain-aging-related research with the ultimate goal of improving the brain health of elderly individuals in both China and the world.

## Introduction

Brain aging refers to the decline in the structure and function of the brain with age, including a decrease in nerve cells, loss of neural network coupling, cognitive decline, mood swings and behavioral changes [1]. Brain aging causes a decline in mental and physical health and increases the risk of age-related brain diseases, such as neurodegenerative diseases. Finding biomarkers that can reflect the level of brain aging can help assess the functional status of the brain and the risk of neurodegenerative diseases in elderly individuals so that timely individualized anti-aging measures can be taken to prevent and treat age-related brain diseases [2].

On 12 March 2023, the Aging Biomarker Consortium (ABC) convened a seminar of experts in brain-aging-related fields at Tongji University. On the basis of literature reports and peer-reviewed domestic research, an expert consensus was formed by combining medical evidence and expert opinions. Biomarkers reflecting brain aging were recommended, with the aim of addressing clinical issues such as ‘How biologically old is the individual now?’, ‘How fast is the individual aging?’, and ‘How far is the individual from age-related brain diseases?’

## Methods

A literature search was conducted for studies published before March 2023 and indexed in MEDLINE, PubMed, Cochrane Library, and other selected databases related to this consensus. For specific search terms used‚ readers are referred to the Online Data Supplement, which contains the final evidence tables summarizing the evidence used by the consensus writing group to formulate recommendations. Members of the ABC first identified a list of key issues related to the biomarkers for brain aging through online collaboration based on available publications and the research of the ABC members. The identified biomarkers were further discussed at a face-to-face meeting to reach a consensus. All recommendations were fully reviewed and discussed among the members of the ABC to allow diverse perspectives and considerations for this consensus.

This consensus follows the internationally accepted conventions for expressing the level of evidence and strength of recommendations, which are detailed in Table 2 [3].

**Table 1.** Abbreviations in this consensus

Abbreviation	Full name
ABC	Aging Biomarker Consortium
COR	Class of recommendation
LOE	Level of evidence
MCI	Mild cognitive impairment
AD	Alzheimer’s disease
TMT	Trail making test
AVLT	Auditory verbal learning test
fMRI	Functional magnetic resonance imaging
MRI	Magnetic resonance imaging
WMH	White matter hyperintensities
TIWI	T1-weighted image
T2WI	T2-weighted image
FLAIR	Fluid-attenuated inversion recovery
SWI	Susceptibility weighted imaging
DTI	Diffusion tensor imaging
DMN	default-mode network
FDG	18F-fluorodeoxyglucose
OCT	Optical coherence tomography
CSF	Cerebrospinal fluid
p-tau	Hyperphosphorylated tau
t-tau	Total tau
NfL	Neurofilament light chain
TREM2	Triggering receptor expressed on myeloid cells 2
sTREM2	Soluble triggering receptor expressed on myeloid cells 2
GFAP	Glial fibrillary acidic protein
SASP	Senescence-associated secretory phenotype
sPDGFRβ	Soluble platelet-derived growth factor receptor beta
ALCAM	Activated leukocyte cell adhesion molecule
FSH	Follicle-stimulating hormone
IGF-1	Insulin-like growth factor 1
NAD	Nicotinamide adenine dinucleotide
NADH	Nicotinamide adenine dinucleotide reduced
GDF11	Growth differentiation factor 11
GAPDH	Reduced glyceraldehyde-phosphate dehydrogenase

**Table 2.** Class of recommendation and level of evidence

Class (Strength) of recommendation	Level (Quality) of evidence
**Class I (Strong) Benefit >>> Risk**	**Level A**
**Suggested phrases for writing recommendation** • Is recommended/is indicated • Evidence and/or general agreement that a given treatment or procedure is beneficial, useful and effective	• Data derived from multiple randomized clinical trials or meta-analyses
**Class IIa (Moderate) Benefit >> Risk**	**Level B**
**Suggested phrases for writing recommendation** • Should be considered • Weight of evidence/opinion is in favor of usefulness/efficacy	• Data derived from a single randomized clinical trial or large non-randomized studies
**Class IIb (Weak) Benefit ≥ Risk**	**Level C**
**Suggested phrases for writing recommendation** • May be considered • Usefulness/efficacy is less well established by evidence/opinion	• Consensus of expert opinion, and/or small studies, retrospective studies, registries
**Class III (Strong) Risk > Benefit**	**Note:** COR and LOE are determined independently (any COR may be paired with any LOE). COR, class of recommendation; LOE, level of evidence.
**Suggested phrases for writing recommendation** • Is not recommended • Evidence/general agreement that the given treatment/procedure is not useful/effective and sometimes maybe harmful	

## Framework of biomarkers for brain aging

**Figure 1.**
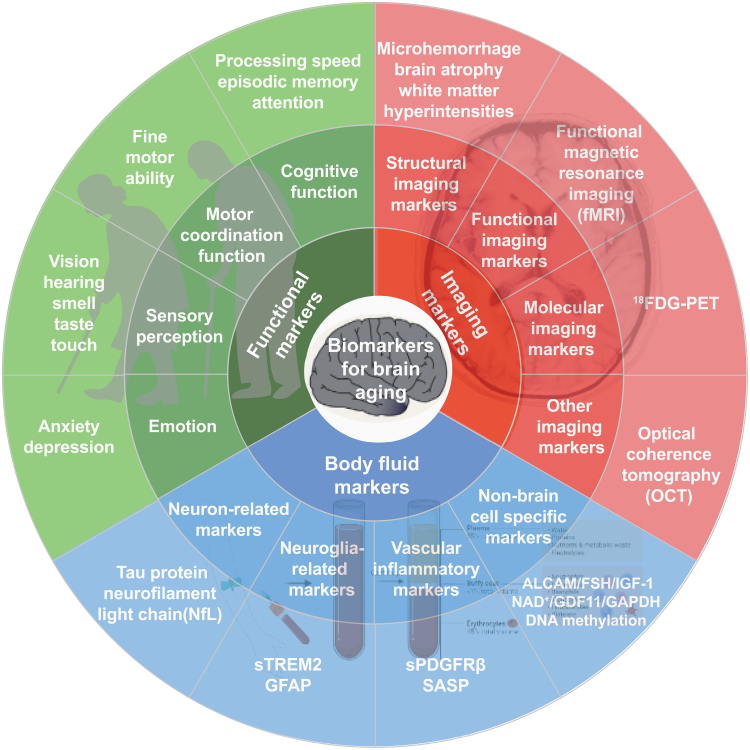
Framework of biomarkers for brain aging. The proposed framework for brain aging is composed of three categories: functional, imaging, and body fluid biomarkers. The recommended biomarkers cover multi-dimensional and multi-scale changes in the brain during aging and are clinically testable. The value of these markers in assessing biological aging of the brain needs further verification.

Brain aging involves multi-dimensional and multi-scale changes in molecules, cells, tissues, organs, the whole body, and groups of individuals. Brain aging biomarkers refer to markers that can accurately reflect ‘real age’, brain structure and brain function, which can be used to determine the degree of brain aging and evaluate the effect of anti-aging interventions. Considering the accessibility and convenience of clinical procedures, this consensus screens brain aging biomarkers from the three dimensions including brain function, imaging, and body fluids for reference in clinical work and follow-up studies (Fig. 1).

### Functional markers

Brain functions include cognitive function, motor coordination, sensory perception, and emotion. These functions are affected by brain aging, but the changes in different brain functions during brain aging are inconsistent. In recent years, studies have begun to explore brain aging biomarkers that are strongly correlated with age, brain structure, and function.

#### Cognitive function

Cognition is the process in which the brain receives, processes, and converts external information into internal mental activities to acquire or apply knowledge, including memory, language, visuospatial processing, executive function, calculation, understanding, and judgment. Behavioral research on aging shows that overall cognitive function declines with increasing age, and the critical time period of decline varies across different cognitive activities [4]. Among these cognitive activities, episodic memory and processing speed decreases are especially effective at revealing the structure and function change of the brain in the aging process and can be used as functional markers to evaluate brain aging.

Episodic memory is the most age-sensitive indicator of cognitive function and declines with age [5–7]. A cohort study involving 306 subjects showed that episodic memory continued to decline during aging and was significantly correlated with the extent of atrophy in the medial temporal lobe and posterior cingulate gyrus [8]. The decline in episodic memory is also closely related to hippocampal atrophy [9] and reduced white matter integrity in elderly individuals [10]. Another small-sample study confirmed that impairment of episodic memory was associated with altered hippocampal-neocortical structural connectivity and an increased risk of developing Alzheimer’s disease (AD) [11]. Functional magnetic resonance imaging (fMRI) studies of episodic memory have shown that the decline in episodic memory encoding performance in elderly individuals is accompanied by increased activation of brain functions in the right prefrontal cortex and orbitofrontal cortex and decreased activation of brain functions in the medial temporal lobe [12, 13]. This suggests that the decline in episodic memory may be related to the decreased connectivity between the prefrontal lobe and the medial temporal lobe [14, 15]. Therefore, episodic memory is closely related to aging and brain structural changes and is a potential biomarker of brain aging. Episodic memory function can be evaluated by cued recall test [16] and an auditory verbal learning test (AVLT) [17]. Coding recognition is a potential tool to evaluate episodic memory and needs to be validated in future studies.

Executive function, a construct that encompasses multiple aspects of cognitive control, exhibits significant age-related differences and may constitute a fundamental feature of human cognitive aging [18]. Attention and processing speed are generally considered to be important components of executive function and are significantly related to aging. Attention refers to the ability to direct and focus mental activities such as perception and thinking toward a specific target. As a multidimensional concept, attention usually includes three functional dimensions: sustained attention, selective attention, and scattered attention. Previous research on attention has shown that while the ability to pay attention to non-task-related information shows a downwards trend with aging [19], the ability to pay attention to task-related information remains unchanged. Therefore, attention cannot be recommended as markers for assessing brain aging.

Processing speed in executive function tasks can be used as a biomarker to predict brain aging. Multiple cross-sectional studies have shown that processing speed decreases linearly with increasing age after 20 years old [20, 21]. A study involving more than 3,000 subjects explored the influence of age on different cognitive domains, and the results suggested that the decline in processing speed with age was the most significant [22]. A meta-analysis that included 91 studies showed a continuous decrease in processing speed with age [23]. Furthermore, the decline in processing speed with age is largely consistent with the volume reduction of the lateral frontal cortex of the brain [24]. A study on the cognitive function of healthy centenarians found that their cognitive function was at a relatively high level, with no significant impairment of processing speed [25]. These results indicate that processing speed can accurately reflect the degree of brain aging and is a potential marker of brain aging. Processing speed can be assessed using the trailmaking test (TMT) [26, 27].

#### Motor coordination function

Physical aging is accompanied by a decline in fine motor skills, which can reflect structural and functional changes in the brain. A study involving more than 3,000 subjects showed that fine motor ability decreased significantly during aging and was significantly correlated with a decrease in gray matter volume [28]. Another study assessing changes in hand fine motor ability during aging showed a continuous decline in this domain with increasing age [29]. Thus, the decline in fine motor ability is highly consistent with aging and changes in brain structure and can be used as a marker of motor function to predict brain aging. Fine motor ability can be assessed by the spiral drawing test and a small pegboard test [28, 29].

#### Sensory perception

Sensory perception includes vision, hearing, smell, taste, and touch. Although the visual and auditory senses decline significantly with age, their dysfunction begins well before the onset of brain aging, due in large part to aging and dysfunction of the peripheral organs themselves, and these senses are, therefore, not suitable as biomarkers of brain aging. The incidence of olfactory impairment increases progressively with age [30]. A cohort study that included 380 subjects showed that olfactory impairment was associated with cognitive decline and brain neurodegeneration [31]. A large cohort study in China showed that the severity of olfactory impairment was significantly associated with the degree of cognitive impairment, increased plasma tau, and neurofilament light chain (NfL) levels, volume reduction in the hippocampus and internal olfactory cortex, and the severity of white matter hyperintensities [32]. Therefore, olfactory dysfunction is associated with brain aging. But it is not recommended as a functional marker of brain aging because of its closer correlation with diseases.

#### Emotion

As the body ages, age-related anxiety also increases significantly and is most common in older women [33]. In clinical samples, the prevalence of anxiety disorders is significantly higher in older adults than in younger adults, with a global estimate of 15% [34]. However, anxiety disorders are no more prevalent in community samples of healthy older adults than in middle-aged adults [34]. In the absence of neurological disorders, the increase in anxiety may be the result of physical decline. Similarly, rodent studies have shown that aging is associated with an increase in depressive behaviors. Geriatric depression usually coexists with general medical and neurological diseases. However, the prevalence of major depressive disorder is much lower in older adults than in younger adults, while approximately 15%–20% of older adults nonetheless present with subclinical depression [35]. Although emotional changes occur during aging, these emotions are mostly secondary to physical frailty and psychological events that are not experienced by all older adults and are therefore not recommended as markers for assessing brain aging.

#### Other functional markers

Language is an important and complex lifelong ability underpinned by dynamic interactions within and between specific networks of the brain. While normal aging compromises specific aspects of language production, most core language processes are robust to brain aging [36]. Frailty syndrome is defined as a state of physiological vulnerability associated with the aging process, resulting in a reduction in homeostatic reserve and difficulty in responding adequately to stressful events [37]. Frailty is a manifestation of various diseases and, in turn, is a risk factor for age-related diseases. Studies have shown that frailty is associated with lower and sharply reduced levels of cognitive function and is most closely related to executive function [38]. As it is influenced by multiple factors, whether frailty can be used as a marker of brain aging needs to be investigated by further studies.

#### Recommendations

- The decrease in processing speed can reflect the degree of brain aging. Clinically, the TMT can be used for evaluation (Level A, Class IIa).
- The decline of episodic memory indicates the possibility of brain aging and an increased risk of developing AD. Episodic memory function can be evaluated by cued recall test and AVLT. (Level B, Class Ⅱa).
- Fine motor ability can be used as a biomarker to reflect brain aging. It can be clinically assessed by the spiral drawing test and a small pegboard test (Level B, Class IIa).

### Imaging markers

Imaging has been widely used to show age-related structural changes in the brain due to its intuitive, accurate, and easy-to-manipulate properties. Increasing evidence shows that structural, functional, and molecular imaging markers can reflect the level of brain aging and predict the risk of age-related brain diseases to varying degrees.

#### Structural imaging markers

##### Brain atrophy

Brain atrophy, defined as morphological changes caused by a decrease in the volume of the brain parenchyma, is one of the most common changes in the elderly brain [39]. A longitudinal follow-up cohort study that included 92 subjects aged 59–85 years found progressive atrophy of brain gray matter with age in healthy elderly people, with frontal and parietal lobe atrophy being the most prominent [40]. A cranial magnetic resonance imaging (MRI) study pooling 10,216 subjects aged 20–90 years from 11 cohorts showed that the frontal lobe, parietal lobe, superior temporal cortex, and insula had the most pronounced atrophy with age in a normal aging population and that this atrophy was significantly associated with executive function decline. For patients with mild cognitive impairment (MCI) or early AD, brain atrophy is mainly manifested as atrophy of the hippocampus, amygdala, entorhinal cortex, and inferior temporal cortex [41, 42]. These results suggest that the pattern of physiological brain atrophy differs from that of pathological brain atrophy, with differences in the brain regions where the atrophy begins.

##### White matter hyperintensities

White matter hyperintensities (WMH) are regions of abnormally high T2-weighted images (T2WI) or T2-fluid attenuated inversion recovery (FLAIR) signal strength in the white matter that is believed to be associated with vascular changes and ischemia in the aging brain. White matter hyperintensities are a common imaging manifestation of brain aging, with a prevalence of more than 85% in people over 60 years of age and a dramatic increase in overall load with age. A craniocerebral imaging study of 10,216 subjects aged 20–90 years found that in a normal aging population, cerebral white matter hyperintensities gradually appeared after the age of 50 years, and their severity was significantly associated with cognitive dysfunction, executive function decline, and amyloid levels in the brain [43]. A large sample study confirmed that cerebral white matter hyperintensities showed a significant correlation with age-related brain atrophy in a healthy aging population [43]. A cohort study that included 491 subjects confirmed that the severity of white matter hyperintensities was negatively correlated with visual executive function and could reflect age-related neurovascular health [44].

##### Cerebral microhemorrhage

With increasing age, the incidence of cerebral microhaemorrhage also increases gradually. The Rotterdam Community Population Study, which included 4,759 subjects followed for an average of 4.9 years, showed an increased risk of stroke with microbleeds at baseline [45]. A small-sample cohort study showed that a cerebral microbleed counts greater than 4 represented diffuse vascular and neurodegenerative injuries, which increased the risk of cognitive decline and dementia in individuals [46]. Therefore, cerebral microhemorrhage is closely related to disease.

#### Functional imaging markers

fMRI reflects neural network activity in the brain. Studies have identified changes associated with normal aging and age-related cognitive impairment. When a person closes their eyes and stops interacting with their surroundings, activity in neural networks, including the precuneus, posterior cingulate cortex, medial prefrontal cortex, and angular gyrus, increases. This ‘default-mode network’ (DMN) is thought to play an important role in recalling the past, thinking about the future and ‘mind wandering’. Reduced functional connectivity of the DMN occurs in the normal aging process and is significantly aggravated in cognitively impaired elderly individuals [47, 48]. fMRI analysis found that the frontoparietal network and its attached neural networks were active in young people and inactive in elderly people in the resting state, and these changes in elderly people were associated with reduced grey matter volume and white matter integrity [49]. In elderly individuals, functional connectivity between the DMN and hippocampus is related to memory ability as measured by performance on memory tasks [50]. Functional connections identified by fMRI analysis have been applied to construct a criterion for ‘brain age’ (see below).

#### Molecular imaging markers

^18^FDG-PET: Reduced brain glucose metabolism is a manifestation of brain aging, and ^18^F-fluorodeoxyglucose (FDG) PET imaging is the primary imaging basis for measuring brain glucose metabolism. A cohort study that included 120 healthy volunteers showed that ^18^F-FDG uptake in the frontal lobes was significantly reduced during normal aging [51]. Another cohort study with a sample size of 123 healthy frontal and inferior parietal cortices with age, which is consistent with structural imaging findings showing significant frontal and parietal atrophy during normal aging [52]. A Japanese study that included 139 healthy volunteers found that ^18^F-FDG metabolism in the brain regions around the medial frontal lobe and lateral sulcus was significantly reduced with age, and this difference was no longer significant after correction by brain atrophy, suggesting that the decrease in ^18^FDG uptake with age as detected by ^18^F-FDG PET is mainly due to age-related brain volume loss [53]. In addition, it has been demonstrated that patients with cognitive decline have reduced metabolism in the posterior cingulate cortex and temporoparietal lobe compared to normal elderly people, and this difference may predict cognitive progression and the risk of conversion to AD during aging [54].

#### Other imaging markers

Optical coherence tomography (OCT) is a new three-dimensional laminar imaging technique that was first used in the field of ophthalmology. In recent years, as the technology has matured, OCT has been gradually applied to the coronary artery and intracranial fields. Animal studies have shown that OCT can reflect the structure and function of cerebral vessels related to aging [55]. Whether OCT can be used as a marker of brain aging needs to be explored and verified by conducting a large number of clinical studies.

#### Recommendations

- Brain atrophy can be used as an imaging marker to assess brain aging, with frontal and parietal atrophy predicting the level of brain aging, while temporal lobe and hippocampal atrophy suggest a potential risk of developing AD. Clinical assessment can be performed by cranial MRI (Level B, Class I).
- The severity of white matter hyperintensities correlates with age and brain function decline and can be used as an imaging marker to assess brain aging. It can be evaluated using FLAIR sequences and diffusion tensor imaging sequences of MRI (Level B, Class I).
- ^18^FDG-PET can be used as an imaging marker to assess brain aging. Decreased ^18^F-FDG uptake in the frontal lobe suggests brain aging and predicts an elevated risk of cognitive impairment; decreased metabolism in the posterior cingulate gyrus and temporoparietal lobe suggests an elevated risk of cognitive decline and development of AD (Level B, Class I).

### Body fluid markers

Components of body fluids such as plasma, urine, and cerebrospinal fluid (CSF) are indispensable biomarkers for the assessment of brain aging due to their noninvasive or minimally invasive collection, high sensitivity, and ease of accurate measurement [56]. This consensus aims to recommend brain aging-related markers; therefore, the search strategy for body fluid markers is to screen body fluids for brain-cell-specific markers or biomarkers that are likely to be highly correlated with brain aging levels. Since these markers also change in various acute and chronic brain diseases, interference from brain diseases should be excluded when the markers are used as brain aging markers.

#### Neuron-related markers

##### Tau protein

Tau protein is a highly soluble microtubule-associated protein that is mainly distributed in the axons of neurons. Under pathological conditions, the normal tau protein in neurons is hyperphosphorylated (p-tau) and aggregated to form neurofibrillary tangles. Elevated p-tau levels in CSF and blood are indirect markers of tau pathology in neurodegenerative diseases, which increase with disease progression and are associated with an increase in neurofibrillary tangles. Studies have found that plasma levels of p-tau and total tau (t-tau) both increase with age, and their increase is associated with reduced cognitive levels and an accelerated rate of cognitive decline [57]. Another small-sample study confirmed that elevated plasma t-tau levels were associated with widespread reductions in cortical glucose uptake, temporal lobe thinning, and memory decline [58]. A longitudinal follow-up cohort study of 276 cognitively normal older adults showed that elevated plasma t-tau levels were positively associated with atrophy of the basal forebrain cholinergic system and could predict the risk of developing AD [59]. Therefore, plasma t-tau and p-tau levels can be used as biological markers to predict brain aging, but care must be taken to differentiate aging from tauopathies.

##### Neurofilament light chain

NfL is a structural protein of neuronal cells that can be detected in body fluids and is a potential biomarker of brain aging and longevity. A large cohort study suggested that plasma NfL increases nonlinearly with age and predicts cognitive decline [60]. Another study found that high levels of plasma NfL were associated with reduced hippocampal volume and the severity of leukoencephalopathy [61]. A cross-sectional study of 335 subjects showed that increased plasma NfL was associated with decreased brain volume in elderly patients [62]. Therefore, plasma NfL levels can be used as a body fluid biomarker to assess brain aging.

#### Microglia-related markers

Triggering receptor expressed on myeloid cells 2 (TREM2) is a transmembrane receptor that plays an important role in regulating microglial phagocytosis and the response to inflammatory stimuli. The extracellular structural domain of TREM2 can be cleaved to produce soluble TREM2 (sTREM2) and released into the CSF and subsequently the blood, serving as an indicator of microglial activity. A cross-sectional study involving 150 subjects showed that CSF and plasma sTREM2 levels were positively correlated with age and significantly correlated with t-tau and p-tau levels in CSF [63]. Another clinical study showed that plasma sTREM2 was correlated with the severity of white matter hyperintensities [64]. Studies have also confirmed that sTREM2 levels in plasma and CSF can predict the risk that MCI will progress to AD [65, 66]. Therefore, plasma and CSF sTREM2 may be considered for use as body fluid markers of brain aging.

#### Astrocyte-related markers

Glial fibrillary acidic protein (GFAP) is a signature intermediate filament in astrocytes that contributes to synaptic formation and axon metabolic homeostasis in the central nervous system. Currently, plasma GFAP is mainly used for the assessment and identification of traumatic brain injury, CNS demyelinating diseases and neurodegenerative diseases [67–69]. Animal studies have shown that plasma GFAP levels increase with age [70, 71]. A meta-analysis based on brain tissue transcriptome datasets showed that the decline in synaptic transmission function and the increase in plasma GFAP were most significant with age [72]. A cross-sectional study of 114 healthy subjects and AD patients confirmed that plasma GFAP levels were significantly associated with the degree of cognitive impairment and white matter lesions [73]. In conclusion, plasma GFAP levels may be considered for use as a body fluid biomarker for the assessment of brain aging, but aging must be distinguished from related diseases.

#### Senescence-associated secretory phenotype molecules

The senescence-associated secretory phenotype (SASP) refers to the proinflammatory factors secreted by cellular senescence, including YKL-40, TNF-α, and IL-1β, which can cause chronic low-grade inflammatory responses and may have an impact on brain aging. Previous studies have shown that the SASP is associated with neuronal apoptosis, decreased synaptic function, and cognitive impairment [74]. However, because these inflammatory markers are nonspecific and are highly influenced by inflammatory diseases and multiple brain diseases, they are not suitable to use alone as brain aging markers.

#### Cerebrovascular markers

CSF levels of soluble platelet-derived growth factor receptor beta (sPDGFRβ), a marker of pericyte damage, increase from 20 years of age onward and are associated with blood-brain barrier dysfunction, dysfunction of neurovascular coupling, and cognitive decline. This molecule can be used as a cerebrovascular aging marker to provide direction for the study of brain aging mechanisms [75].

#### Non-brain-cell-specific markers

Activated leukocyte cell adhesion molecule (ALCAM) is a transmembrane immunoglobulin that is involved in cellular immunity and neuroinflammatory processes. A peer-reviewed domestic cohort study of aging has shown that plasma ALCAM levels are significantly correlated with brain aging, and this needs to be further verified in future cohort studies. Multiple studies have confirmed that plasma follicle-stimulating hormone (FSH), insulin-like growth factor 1 (IGF-1), nicotinamide adenine dinucleotide (NAD^+^) or NAD^+^/ nicotinamide adenine dinucleotide reduced (NADH), GDF11 and GAPDH mRNA, and DNA methylation levels are significantly correlated with overall aging [76–80]. However, there is a lack of relevant clinical evidence indicating whether they can be used as specific markers to assess the level of brain aging. These markers may be considered for use as brain aging biomarkers to be validated in follow-up cohort studies.

#### Recommendations

- Plasma t-tau and p-tau levels may be useful body fluid biomarkers to predict brain aging, and their elevation suggests the possibility of brain aging and an increased risk of neurodegenerative diseases (Level B, Class IIa).
- Plasma NfL levels may be a useful body fluid biomarker to predict brain aging, and increased NfL levels suggest the possibility of brain aging and the risk of cognitive decline (Level B, Class IIa).
- Plasma and CSF sTREM2 may be considered for use as body fluid biomarkers to assess brain aging (Level B, Class IIa).
- Plasma and CSF GFAP may be considered for use as body fluid biomarkers for the assessment of brain aging (Level B, Class IIa).
- (v) Plasma ALCAM, FSH, IGF-1, NAD^+^ or NAD^+^/NADH, GDF11 and GAPDH mRNA, and DNA methylation levels may be considered for use as candidate biomarkers of brain aging, but should be validated in follow-up cohort studies (Level C, Class IIb).

### Brain age model

Brain aging involves changes in all aspects of the brain, from molecular to functional levels. Therefore, multi-dimensional and multi-scale information needs to be integrated to accurately evaluate the state of brain aging. In recent years, remarkable progress has been made in exploring the biological age of the brain.

#### Brain age assessment model

Biological age is the age deduced according to the developmental state of normal human physiology and anatomy, so as to accurately reflect the actual state of human organization structure and physiological function. Biological age can provide a more accurate quantitative index than chronological age for evaluating the senescence of the human body and answering the scientific question of age in physiological terms. The abovementioned brain aging biomarkers provide options for the construction of biological age of the brain (hereinafter referred to as ‘brain age'), and conversely, brain age can provide a reference standard for the study of brain aging markers. In addition, brain age can provide a basis for research on the mechanisms of brain aging, a method for early identification of pathological aging, and a basis for early intervention and efficacy evaluation. The rise of machine learning has promoted research on ‘brain age assessment models’. Currently, the construction of brain age is mostly based on structural imaging and electroencephalography [81–83]. With the development of functional imaging, functional connectivity (FC) and multi-modal imaging (sMRI+FC) have been gradually applied to the construction of brain age [84, 85]. These brain age models are currently evaluated based on the difference between predicted and actual brain age, that is, the brain-age gap (BAG), which is usually between 1 and 10 years. However, the current brain-age models lack external validation in different cohorts or centers.

#### Brain aging and age-related brain disease prediction model

The BAG reflects whether the aging of the brain is accelerated or delayed, laying a foundation for the prediction of age-related brain diseases. It can accurately predict how fast an individual is aging and how far an individual is from age-related brain diseases. To date, BAG has been used to predict a variety of brain diseases, including AD, MCI, epilepsy, and psychiatric diseases. Studies have found that positive BAG or increased levels of BAG are associated with the risk of cognitive impairment, traumatic brain injury, and schizophrenia [82]. It should be noted that the prediction of brain age cannot be used as an independent predictor of disease due to its lack of specificity; therefore, disease-specific markers should be combined. Current predictive models of brain age are based on cross-sectional studies; therefore, there is a lack of effective comparison and verification with longitudinal follow-up data for prognostic assessment.

The aging process involves multi-scale and multi-dimensional changes in molecules, cells, organs, and the whole body, and indicators on different scales convey different information. The establishment of brain age models and age-related brain disease prediction models based on multi-scale, multi-dimensional, and multi-modal markers may be more accurate, stable, and universal, and research is moving in this direction.

## Conclusion and future perspectives

According to the experts’ discussion, the brain aging markers are recommended in the three dimensions of behavioral function, imaging, and body fluids; these markers will be further validated in different age groups in the future (Table 3).

**Table 3.** Recommended biomarkers of brain aging

Dimension	Biomarker	Test method	COR	LOE
Functional markers	Processing speed	Trail making test (TMT)	**IIa**	**A**
	Episodic memory	Cued recall test and auditory verbal learning test (AVLT)	**IIa**	**B**
	Fine motor ability	Spiral drawing test and small pegboard test	**IIa**	**B**
Imaging markers	Brain atrophy	MRI	**I**	**B**
	White matter hyperintensities	MRI using FLAIR and DTI sequences	**I**	**B**
	^18^FDG uptake	^18^FDG-PET	**I**	**B**
Body fluid markers	t-tau/p-tau	Plasma/ELISA	**IIa**	**B**
	NfL	Plasma/ELISA	**IIa**	**B**
	sTREM2	Plasma/ELISA	**IIa**	**B**
	GFAP	Plasma/ELISA	**IIa**	**B**
	ALCAM*	Plasma/ELISA	**IIb**	**C**
	IGF-1*	Plasma/ELISA	**IIb**	**C**
	FSH*	Plasma/ELISA	**IIb**	**C**
	NAD^+^*	Plasma/mass spectrometry	**IIb**	**C**
	GDF11/GAPDH*	Plasma/qRTPCR	**IIb**	**C**
	DNA methylation*	Plasma/methylation PCR array	**IIb**	**C**

^*^These indicators lack clinical evidence related to brain aging and need to be validated in future cohort studies.

The working road map of brain aging biomarker research in China includes the following goals: (i) To establish a national multicentre aging cohort of approximately 1000 subjects to discover and validate brain aging biomarkers, establish detection techniques and methods, determine the reference values of brain aging biomarkers in the Chinese population, predict the ‘turning point’ of brain aging and determine the time window for intervention. (ii) To establish a brain aging assessment model and age-related brain disease prediction model by using artificial intelligence calculations. (iii) To establish a research and development paradigm combining industry, academia, research, and government to promote the translation and application of research achievements. Establishment of the framework of biomarkers for brain aging will also facilitate the research for neurodegenerative diseases for which aging is a major risk factor. The ultimate goal is to improve the level of brain health of the elderly and achieve healthy aging worldwide.

## References

[1] Cai Y, Song W, Li J, et al. The landscape of aging. Sci China Life Sci 2022;65:2354–454. 36066811 10.1007/s11427-022-2161-3 PMC9446657

[2] Aging Biomarker C et al. Biomarkers of aging. Sci China Life Sci 2023;66(5):893–1066. 37076725 10.1007/s11427-023-2305-0 PMC10115486

[3] Sousa-Uva M, Head SJ, Thielmann M, et al. Methodology manual for European Association for Cardio-Thoracic Surgery (EACTS) clinical guidelines. Eur J Cardiothorac Surg 2015;48:809–16. 26362426 10.1093/ejcts/ezv309

[4] Li H, Lv C, Zhang T, et al. Trajectories of age-related cognitive decline and potential associated factors of cognitive function in senior citizens of Beijing. Curr Alzheimer Res 2014;11:806–16. 25212920 10.2174/156720501108140910123112

[5] Tromp D, Dufour A, Lithfous S, et al. Episodic memory in normal aging and Alzheimer disease: insights from imaging and behavioral studies. Ageing Res Rev 2015;24:232–62. 26318058 10.1016/j.arr.2015.08.006

[6] Nyberg L. Functional brain imaging of episodic memory decline in ageing. J Intern Med 2017;281:65–74. 27453565 10.1111/joim.12533

[7] Lempert KM, Cohen MS, MacNear KA, et al. Aging is associated with maladaptive episodic memory-guided social decision-making. Proc Natl Acad Sci U S A 2022;119:e2208681119. 36215461 10.1073/pnas.2208681119 PMC9586277

[8] Pelletier A, Bernard C, Dilharreguy B, et al. Patterns of brain atrophy associated with episodic memory and semantic fluency decline in aging. Aging (Albany NY) 2017;9:741–52. 28278492 10.18632/aging.101186 PMC5391228

[9] Persson J, Nyberg L, Lind J, et al. Structure-function correlates of cognitive decline in aging. Cereb Cortex 2006;16:907–15. 16162855 10.1093/cercor/bhj036

[10] Charlton RA, Barrick TR, Markus HS, et al. The relationship between episodic long-term memory and white matter integrity in normal aging. Neuropsychologia 2010;48:114–22. 19699215 10.1016/j.neuropsychologia.2009.08.018

[11] Ousdal OT, Kaufmann T, Kolskår K, et al. Longitudinal stability of the brain functional connectome is associated with episodic memory performance in aging. Hum Brain Mapp 2020;41:697–709. 31652017 10.1002/hbm.24833 PMC7268077

[12] Nyberg L, Sandblom J, Jones S, et al. Neural correlates of training-related memory improvement in adulthood and aging. Proc Natl Acad Sci U S A 2003;100:13728–33. 14597711 10.1073/pnas.1735487100 PMC263881

[13] Dennis NA, Daselaar S, Cabeza R. Effects of aging on transient and sustained successful memory encoding activity. Neurobiol Aging 2007;28:1749–58. 16919850 10.1016/j.neurobiolaging.2006.07.006 PMC3691865

[14] Grady CL, McIntosh AR, Horwitz B, et al. Age-related reductions in human recognition memory due to impaired encoding. Science 1995;269:218–21. 7618082 10.1126/science.7618082

[15] Wang L, Li H, Liang Y, et al. Amnestic mild cognitive impairment: topological reorganization of the default-mode network. Radiology 2013;268:501–14. 23481166 10.1148/radiol.13121573

[16] Benejam B, Aranha MR, Videla L, et al. Neural correlates of episodic memory in adults with Down syndrome and Alzheimer’s disease. Alzheimers Res Ther 2022;14:123. 36057615 10.1186/s13195-022-01064-x PMC9440567

[17] Moradi E, Hallikainen I, Hänninen T, et al; Alzheimer's Disease Neuroimaging Initiative. Rey’s Auditory Verbal Learning Test scores can be predicted from whole brain MRI in Alzheimer’s disease. Neuroimage Clin 2017;13:415–27. 28116234 10.1016/j.nicl.2016.12.011 PMC5233798

[18] Lacreuse A, Raz N, Schmidtke D, et al. Age-related decline in executive function as a hallmark of cognitive ageing in primates: an overview of cognitive and neurobiological studies. Philos Trans R Soc Lond B Biol Sci 2020;375:20190618. 32951543 10.1098/rstb.2019.0618 PMC7540957

[19] Geerligs L, Saliasi E, Maurits NM, et al. Brain mechanisms underlying the effects of aging on different aspects of selective attention. Neuroimage 2014;91:52–62. 24473095 10.1016/j.neuroimage.2014.01.029

[20] Zelinski EM, Burnight KP. Sixteen-year longitudinal and time lag changes in memory and cognition in older adults. Psychol Aging 1997;12:503–13. 9308097 10.1037//0882-7974.12.3.503

[21] Park DC, Lautenschlager G, Hedden T, et al. Models of visuospatial and verbal memory across the adult life span. Psychol Aging 2002;17:299–320. 12061414

[22] Hoogendam YY, Hofman A, van der Geest JN, et al. Patterns of cognitive function in aging: the Rotterdam Study. Eur J Epidemiol 2014;29:133–40. 24553905 10.1007/s10654-014-9885-4

[23] Verhaeghen P, Salthouse TA. Meta-analyses of age-cognition relations in adulthood: estimates of linear and nonlinear age effects and structural models. Psychol Bull 1997;122:231–49. 9354147 10.1037/0033-2909.122.3.231

[24] Elliott ML, Belsky DW, Knodt AR, et al. Brain-age in midlife is associated with accelerated biological aging and cognitive decline in a longitudinal birth cohort. Mol Psychiatry 2021;26:3829–38. 31822815 10.1038/s41380-019-0626-7 PMC7282987

[25] Beker N, Ganz A, Hulsman M, et al. Association of cognitive function trajectories in centenarians with postmortem neuropathology, physical health, and other risk factors for cognitive decline. JAMA Netw Open 2021;4:e2031654. 33449094 10.1001/jamanetworkopen.2020.31654 PMC7811180

[26] Llinàs-Reglà J, Vilalta-Franch J, López-Pousa S, et al. The trail making test. Assessment 2017;24:183–96. 26318386 10.1177/1073191115602552

[27] Fett AJ, Velthorst E, Reichenberg A, et al. Long-term changes in cognitive functioning in individuals with psychotic disorders: findings from the Suffolk county mental health project. JAMA Psychiatry 2020;77:387–96. 31825511 10.1001/jamapsychiatry.2019.3993 PMC6990826

[28] Hoogendam YY, van der Lijn F, Vernooij MW, et al. Older age relates to worsening of fine motor skills: a population-based study of middle-aged and elderly persons. Front Aging Neurosci 2014;6:259–265. 25309436 10.3389/fnagi.2014.00259 PMC4174769

[29] Quandt F, Bönstrup M, Schulz R, et al. Spectral variability in the aged brain during fine motor control. Front Aging Neurosci 2016;8:305. 28066231 10.3389/fnagi.2016.00305 PMC5175385

[30] Ferdon S, Murphy C. The cerebellum and olfaction in the aging brain: a functional magnetic resonance imaging study. Neuroimage 2003;20:12–21. 14527566 10.1016/s1053-8119(03)00276-3

[31] Dintica CS, Marseglia A, Rizzuto D, et al. Impaired olfaction is associated with cognitive decline and neurodegeneration in the brain. Neurology 2019;92:e700–9. 30651382 10.1212/WNL.0000000000006919 PMC6382360

[32] Dong Y, Li Y, Liu K, et al. Anosmia, mild cognitive impairment, and biomarkers of brain aging in older adults. Alzheimers Dement 2023;19:589–601. 36341691 10.1002/alz.12777

[33] Volkert J, Schulz H, Härter M, et al. The prevalence of mental disorders in older people in Western countries—a meta-analysis. Ageing Res Rev 2013;12:339–53. 23000171 10.1016/j.arr.2012.09.004

[34] AM NM et al. Frailty, depression, and anxiety in later life. Int Psychogeriatr 2012;24:1265–74. 22333477 10.1017/S1041610211002110

[35] Prenderville JA, Kennedy PJ, Dinan TG, et al. Adding fuel to the fire: the impact of stress on the ageing brain. Trends Neurosci 2015;38:13–25. 25705750 10.1016/j.tins.2014.11.001

[36] Shafto MA, Tyler LK. Language in the aging brain: the network dynamics of cognitive decline and preservation. Science 2014;346:583–7. 25359966 10.1126/science.1254404

[37] Fabricio DM, Chagas MHN, Diniz BS. Frailty and cognitive decline. Transl Res 2020;221:58–64. 32045578 10.1016/j.trsl.2020.01.002

[38] Chu NM, Xue Q-L, McAdams-DeMarco MA, et al. Frailty-a risk factor of global and domain-specific cognitive decline among a nationally representative sample of community-dwelling older adult U.S. Medicare beneficiaries. Age Ageing 2021;50:1569–77. 34097002 10.1093/ageing/afab102 PMC8437073

[39] Huang P, Zhang M. Magnetic resonance imaging studies of neurodegenerative disease: from methods to translational research. Neurosci Bull 2023;39:99–112. 35771383 10.1007/s12264-022-00905-x PMC9849544

[40] Kakimoto A, Ito S, Okada H, et al. Age-related sex-specific changes in brain metabolism and morphology. J Nucl Med 2016;57:221–5. 26609179 10.2967/jnumed.115.166439

[41] Pini L, Pievani M, Bocchetta M, et al. Brain atrophy in Alzheimer’s disease and aging. Ageing Res Rev 2016;30:25–48. 26827786 10.1016/j.arr.2016.01.002

[42] Habes M, Pomponio R, Shou H, et al; iSTAGING consortium, the Preclinical AD consortium, the ADNI, and the CARDIA studies. The Brain Chart of Aging: machine-learning analytics reveals links between brain aging, white matter disease, amyloid burden, and cognition in the iSTAGING consortium of 10,216 harmonized MR scans. Alzheimers Dement 2021;17:89–102. 32920988 10.1002/alz.12178 PMC7923395

[43] Habes M, Erus G, Toledo JB, et al. White matter hyperintensities and imaging patterns of brain ageing in the general population. Brain 2016;139:1164–79. 26912649 10.1093/brain/aww008 PMC5006227

[44] Huang CC, Chou K-H, Lee W-J, et al. Brain white matter hyperintensities-predicted age reflects neurovascular health in middle-to-old aged subjects. Age Ageing 2022;51:1–10. 10.1093/ageing/afac106 35536881

[45] Montandon ML, Herrmann FR, Garibotto V, et al. Microbleeds and medial temporal atrophy determine cognitive trajectories in normal aging: a longitudinal PET-MRI study. J Alzheimers Dis 2020;77:1431–42. 32925053 10.3233/JAD-200559

[46] Lim JS, Hong K-S, Kim G-M, et al. Cerebral microbleeds and early recurrent stroke after transient ischemic attack: results from the Korean Transient Ischemic Attack Expression Registry. JAMA Neurol 2015;72:301–8. 25580844 10.1001/jamaneurol.2014.3958

[47] Leal SL, Yassa MA. Perturbations of neural circuitry in aging, mild cognitive impairment, and Alzheimer’s disease. Ageing Res Rev 2013;12:823–31. 23380151 10.1016/j.arr.2013.01.006 PMC3893046

[48] Sala-Llonch, R, Bartrés-Faz D, Junqué C. Reorganization of brain networks in aging: a review of functional connectivity studies. Front Psychol 2015;6:663. 26052298 10.3389/fpsyg.2015.00663 PMC4439539

[49] Marstaller L, Williams M, Rich A, et al. Aging and large-scale functional networks: white matter integrity, gray matter volume, and functional connectivity in the resting state. Neuroscience 2015;290:369–78. 25644420 10.1016/j.neuroscience.2015.01.049

[50] Li X, Kehoe EG, McGinnity TM, et al. Modulation of effective connectivity in the default mode network at rest and during a memory task. Brain Connect 2015;5:60–7. 25390185 10.1089/brain.2014.0249

[51] Loessner A, Alavi A, Lewandrowski KU, et al. Regional cerebral function determined by FDG-PET in healthy volunteers: normal patterns and changes with age. J Nucl Med 1995;36:1141–9. 7790936

[52] Yoshizawa H, Gazes Y, Stern Y, et al. Characterizing the normative profile of 18F-FDG PET brain imaging: sex difference, aging effect, and cognitive reserve. Psychiatry Res 2014;221:78–85. 24262800 10.1016/j.pscychresns.2013.10.009

[53] Yanase D, Matsunari I, Yajima K, et al. Brain FDG PET study of normal aging in Japanese: effect of atrophy correction. Eur J Nucl Med Mol Imaging 2005;32:794–805. 15759148 10.1007/s00259-005-1767-2

[54] Pagani M, Giuliani A, Öberg J, et al. Progressive disintegration of brain networking from normal aging to Alzheimer disease: analysis of independent components of (18)F-FDG PET Data. J Nucl Med 2017;58:1132–9. 28280223 10.2967/jnumed.116.184309

[55] Li Y, Choi WJ, Wei W, et al. Aging-associated changes in cerebral vasculature and blood flow as determined by quantitative optical coherence tomography angiography. Neurobiol Aging 2018;70:148–59. 30007164 10.1016/j.neurobiolaging.2018.06.017 PMC6119107

[56] Huang S, Wang YJ, Guo J. Biofluid biomarkers of Alzheimer’s disease: progress, problems, and perspectives. Neurosci Bull 2022;38:677–91. 35306613 10.1007/s12264-022-00836-7 PMC9206048

[57] Chiu MJ, Fan L-Y, Chen T-F, et al. Plasma tau levels in cognitively normal middle-aged and older adults. Front Aging Neurosci 2017;9:51. 28321189 10.3389/fnagi.2017.00051 PMC5337523

[58] Cantero JL, Atienza M, Ramos-Cejudo J, et al. Plasma tau predicts cerebral vulnerability in aging. Aging (Albany NY) 2020;12:21004–22. 33147571 10.18632/aging.104057 PMC7695405

[59] Cavedo, E, Lista S, Houot M, et al., Alzheimer Precision Medicine Initiative; INSIGHT-preAD Study Group. Plasma tau correlates with basal forebrain atrophy rates in people at risk for Alzheimer disease. Neurology 2020;94:e30–41. 31801830 10.1212/WNL.0000000000008696

[60] Kaeser S et al. A neuronal blood marker is associated with mortality in old age. Nature Aging 2021;1:218–225. 37118632 10.1038/s43587-021-00028-4

[61] Dittrich A, Ashton NJ, Zetterberg H, et al. Plasma and CSF NfL are differentially associated with biomarker evidence of neurodegeneration in a community-based sample of 70-year-olds. Alzheimers Dement (Amst) 2022;14:e12295. 35280965 10.1002/dad2.12295 PMC8897823

[62] Khalil M, Pirpamer L, Hofer E, et al. Serum neurofilament light levels in normal aging and their association with morphologic brain changes. Nat Commun 2020;11:812. 32041951 10.1038/s41467-020-14612-6 PMC7010701

[63] Henjum K, Almdahl IS, Årskog V, et al. Cerebrospinal fluid soluble TREM2 in aging and Alzheimer’s disease. Alzheimers Res Ther 2016;8:17. 27121148 10.1186/s13195-016-0182-1 PMC4848774

[64] Tsai HH, Chen Y-F, Yen R-F, et al. Plasma soluble TREM2 is associated with white matter lesions independent of amyloid and tau. Brain 2021;144:3371–80. 34515756 10.1093/brain/awab332

[65] Park SH, Lee E-H, Kim H-J, et al. The relationship of soluble TREM2 to other biomarkers of sporadic Alzheimer’s disease. Sci Rep 2021;11:13050. 34158530 10.1038/s41598-021-92101-6 PMC8219697

[66] Zhao A, Jiao Y, Ye G, et al. Soluble TREM2 levels associate with conversion from mild cognitive impairment to Alzheimer’s disease. J Clin Invest 2022;132:1–11. 10.1172/JCI158708 PMC9753995 36519540

[67] Abdelhak A, Hottenrott T, Morenas-Rodríguez E, et al. Glial activation markers in CSF and serum from patients with primary progressive multiple sclerosis: potential of serum GFAP as disease severity marker? Front Neurol 2019;10:280. 30972011 10.3389/fneur.2019.00280 PMC6443875

[68] Abdelhak A, Foschi M, Abu-Rumeileh S, et al. Blood GFAP as an emerging biomarker in brain and spinal cord disorders. Nat Rev Neurol 2022;18:158–72. 35115728 10.1038/s41582-021-00616-3

[69] Korley FK, Jain S, Sun X, et al; TRACK-TBI Study Investigators. Prognostic value of day-of-injury plasma GFAP and UCH-L1 concentrations for predicting functional recovery after traumatic brain injury in patients from the US TRACK-TBI cohort: an observational cohort study. Lancet Neurol 2022;21:803–13. 35963263 10.1016/S1474-4422(22)00256-3 PMC9462598

[70] Krekoski CA, Parhad IM, Fung TS, et al. Aging is associated with divergent effects on Nf-L and GFAP transcription in rat brain. Neurobiol Aging 1996;17:833–41. 9363793 10.1016/s0197-4580(96)00078-4

[71] Anderson CP, Rozovsky I, Stone DJ, et al. Aging and increased hypothalamic glial fibrillary acid protein (GFAP) mRNA in F344 female rats. Dissociation of GFAP inducibility from the luteinizing hormone surge. Neuroendocrinology 2002;76:121–30. 12169773 10.1159/000064429

[72] Wruck W, Adjaye J. Meta-analysis of human prefrontal cortex reveals activation of GFAP and decline of synaptic transmission in the aging brain. Acta Neuropathol Commun 2020;8:26. 32138778 10.1186/s40478-020-00907-8 PMC7059712

[73] Bettcher BM, Olson KE, Carlson NE, et al. Astrogliosis and episodic memory in late life: higher GFAP is related to worse memory and white matter microstructure in healthy aging and Alzheimer’s disease. Neurobiol Aging 2021;103:68–77. 33845398 10.1016/j.neurobiolaging.2021.02.012 PMC8313091

[74] Han X, Lei Q, Xie J, et al. Potential regulators of the senescence-associated secretory phenotype during senescence and aging. J Gerontol A Biol Sci Med Sci 2022;77:2207–18. 35524726 10.1093/gerona/glac097

[75] Wang P, Yu Le, Gong J, et al. An activity-based fluorescent probe for imaging fluctuations of peroxynitrite (ONOO(-)) in the Alzheimer’s disease brain. Angew Chem Int Ed Engl 2022;61:e202206894. 35789171 10.1002/anie.202206894

[76] Ono T, Uehara Y, Kurishita A, et al. Biological significance of DNA methylation in the ageing process. Age Ageing 1993;22:S34–43. 8438654 10.1093/ageing/22.suppl_1.s34

[77] Junnila RK, List EO, Berryman DE, et al. The GH/IGF-1 axis in ageing and longevity. Nat Rev Endocrinol 2013;9:366–76. 23591370 10.1038/nrendo.2013.67 PMC4074016

[78] Liu XM, Chan HC, Ding G-L, et al. FSH regulates fat accumulation and redistribution in aging through the Gαi/Ca(2+)/CREB pathway. Aging Cell 2015;14:409–20. 25754247 10.1111/acel.12331 PMC4406670

[79] Schafer MJ, Atkinson EJ, Vanderboom PM, et al. Quantification of GDF11 and myostatin in human aging and cardiovascular disease. Cell Metab 2016;23:1207–15. 27304512 10.1016/j.cmet.2016.05.023 PMC4913514

[80] Crunkhorn S. Aging: promoting NAD(+) production. Nat Rev Drug Discov 2018;17:864. 10.1038/nrd.2018.212 30482958

[81] Paixao L, Sikka P, Sun H, et al. Excess brain age in the sleep electroencephalogram predicts reduced life expectancy. Neurobiol Aging 2020;88:150–5. 31932049 10.1016/j.neurobiolaging.2019.12.015 PMC7085452

[82] Baecker L, Garcia-Dias R, Vieira S, et al. Machine learning for brain age prediction: introduction to methods and clinical applications. EBioMedicine 2021;72:103600. 34614461 10.1016/j.ebiom.2021.103600 PMC8498228

[83] Yook S, Park HR, Park C, et al. Novel neuroelectrophysiological age index associated with imaging features of brain aging and sleep disorders. Neuroimage 2022;264:119753. 36400380 10.1016/j.neuroimage.2022.119753

[84] Liem F, Varoquaux G, Kynast J, et al. Predicting brain-age from multimodal imaging data captures cognitive impairment. Neuroimage 2017;148:179–88. 27890805 10.1016/j.neuroimage.2016.11.005

[85] Millar PR, Gordon BA, Luckett PH, et al. Multimodal brain age estimates relate to Alzheimer disease biomarkers and cognition in early stages: a cross-sectional observational study. Elife 2023;12:1–25. 10.7554/eLife.81869 PMC9988262 36607335

